# Transthyretin increases migration and invasion of rat placental trophoblast cells

**DOI:** 10.1002/2211-5463.12911

**Published:** 2020-07-01

**Authors:** Xiao‐Peng Ma, Chong‐Dong Liu, Guang‐Ming Cao, Zhen‐Yu Zhang

**Affiliations:** ^1^ Beijing Chaoyang Hospital Affiliated to Capital Medical University Beijing China; ^2^ Beijing Youan Hospital Affiliated to Capital Medical University Beijing China

**Keywords:** biomarker, MMP‐2, MMP‐9, preeclampsia, transthyretin, trophoblast cells

## Abstract

Preeclampsia (PE) is a hypertensive disorder of pregnancy. Early diagnosis of PE is currently contingent on regular prenatal physical examinations and may be facilitated by identification of novel diagnostic markers. Transthyretin (TTR), also known as prealbumin, is primarily responsible for maintaining the normal levels of thyroxine and retinol binding protein. The expression of TTR is lower in patients with severe PE as compared with healthy controls. Here, we examined the suitability of TTR as a diagnostic marker in pregnant hypertensive rats. *N*′‐nitro‐l‐arginine‐methylesterhydrochloride (l‐NAME) was used to generate a rat model of hypertension during pregnancy. Rat placental trophoblast cells were divided into control and TTR groups for *in vitro* experiments. Systolic blood pressure, diastolic blood pressure, mean blood pressure and urinary protein of hypertensive pregnant rats were higher than those of healthy pregnant rats, but these effects could be reversed by TTR treatment. There were no significant changes in blood pressure and urinary protein in healthy pregnant rats before or after TTR treatment. TTR levels in the serum and placental tissues of pregnant hypertensive rats were significantly reduced compared with those of healthy pregnant rats. Changes in placental and fetal weights in the hypertensive model could also be rescued by TTR treatment. TTR treatment significantly increased the level of matrix metalloproteinase‐2/9 in hypertensive rats. Finally, *in vivo* and *in vitro* experiments demonstrated that TTR effectively increased the migration and invasion of rat placental trophoblast cells, as well as matrix metalloproteinase‐2/9 levels in these cells. In conclusion, our data from a rat model suggest that TTR may have potential as a novel marker for PE diagnosis.

## Graphical abstract

Female rats were treated with *N*′‐nitro‐l‐arginine‐methylesterhydrochloride to establish a model of preeclampsia and subsequently treated with transthyretin. Blood pressure, urinary protein, and pathological and molecular changes in the placenta were measured. We also examined cell migration and protein expression in primary trophoblasts and trophoblast cell line JAR.

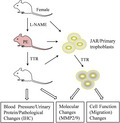

**Abbreviations**
AD: Alzheimer's disease
DBP: diastolic blood pressure
l‐NAME: *N*′‐nitro‐l‐arginine‐methylesterhydrochloride
MBP: mean blood pressure
MMP: matrix metalloproteinase
PE: preeclampsia
SBP: systolic blood pressure
SD: standard deviation
TTR: transthyretin
WB: western blot

Preeclampsia (PE) is a serious hypertensive disorder in pregnancy that causes increased perinatal morbidity and mortality [1]. It is also a relatively difficult clinical problem for obstetricians. At present, it is believed that the pathogenesis of PE is divided into two stages. The first stage is the early clinical stage; that is, remodeling disorder of the uterine spiral arteries induces placental ischemia and hypoxia, which results in the release of placental growth factors. In the second stage, placental growth factors have access to the peripheral circulation and promote the activation of systemic inflammation and the damage of vessel endothelium, thus causing hypertension, edema, proteinuria and other clinical symptoms [2, 3]. PE usually occurs after 20 weeks of gestation, and early diagnosis of PE mainly relies on providing regular prenatal physical examination before delivery [4]. Hence it is urgent to find a new diagnostic marker for PE.

Transthyretin (TTR), also known as prealbumin, is primarily responsible for maintaining the normal levels of thyroxine and retinol binding protein. TTR is able to transport about 15% thyroxine under normal physiological conditions [5, 6]. The abnormal expression of TTR is closely related to many diseases, such as familial amyloidotic polyneuropathy [7], Alzheimer’s disease (AD) [8] and tumors [9, 10]. In addition, serum TTR levels can reflect the nutritional status and some pathophysiological changes in time [11]. Furthermore, compared with healthy pregnant women, the expression of TTR in patients with severe PE is significantly down‐regulated [12]. To further reveal the relationship between TTR and hypertensive disease in pregnancy, the ideal animal model of pregnancy‐induced hypertension is urgently needed. In this study of PE pathogenesis, we used *N′*‐nitro‐l‐arginine‐methylesterhydrochloride (l‐NAME) to establish a hypertensive rat model in pregnancy that can effectively simulate the pathophysiological changes of PE [13]. On the basis of the effective model, the effects of TTR on the pathophysiology of PE and its functions in rat placental trophoblast cells were analyzed.

## Materials and methods

### Animal models

CD‐1 rats (No. 402; Charles River, Beijing, China) were housed in pairs. If sperm were observed in vaginal secretions of female rats, the day would be recorded as day 0 of pregnancy. Pregnant rats were randomly divided into two groups: healthy pregnancy group (control group) and pregnancy‐induced hypertension model group (l‐NAME group, 24 rats). The rats in the l‐NAME group were subcutaneously injected with l‐NAME (50 mg·kg^−1^·day^−1^) on the 7th to 11th day of pregnancy. The rats in the control group (*n* = 8) were injected with saline. All animal experiments were performed according to Guide for Ethical Review of Animal Welfare (GB/T35892‐2018, China) and approved by the Ethics Committee of Beijing Chaoyang Hospital Affiliated to Capital Medical University.

### TTR treatment

TTR, purchased from PeproTech (Rocky Hill, NJ, USA), was derived from human recombinant TTR protein. After the establishment of pregnant hypertension models, the control group or l‐NAME group was randomly divided into two groups. One group was intraperitoneally injected with TTR (100 μg) on the 11th day of gestation [TTR group (eight rats) or l‐NAME + TTR group (nine rats)]; the other group was injected with saline [control group (9 rats) or l‐NAME group (11 rats)]. After that, the changes of blood pressure and urine protein of pregnant rats in each group were monitored, respectively. The urine protein content of rats was measured by the sulfosalicylic acid method. The specific detection procedures were shown in the published article [14]. The tail‐cuff method was used to measure blood pressure in rats [15]. Rats were sacrificed after 18 days of pregnancy, and the weights of placenta and fetal rats in each group were analyzed.

### ELISA

On the 18th day of pregnancy, blood samples were collected from postorbital blood vessels of pregnant rats. The blood samples were centrifuged for 15 min (448 ***g***). Serum TTR levels were determined by ELISA (ab108904; Abcam, Cambridge, MA, USA) according to the manufacturer’s instructions.

### Western blot analysis

The protein lysates were separated by 10% SDS/PAGE and then transferred onto polyvinylidene fluoride membranes. Then, the blots were blocked in 5% nonfat milk for 1 h and incubated at 4 °C overnight with the primary antibody: anti‐TTR Ig (MFCD00162547; Sigma, St Louis, MO, USA), anti‐matrix metalloproteinase‐2 (anti‐MMP2) Ig (ab37150; Abcam), or anti‐MMP9 Ig (ab38898; Abcam).

### Cell culture

Trophoblast cell line JAR was purchased from the Institute of Microbiology, Chinese Academy of Sciences. Cells were cultured in Dulbecco’s modified Eagle’s medium (#12634‐010; Thermo Scientific, Waltham, MA, USA) containing 5% FBS (SH30070.02; HyClone, Logan, UT, USA) at 37 °C in an incubator with 5% CO_2_.

### Transwell assays

Cell migration and invasion were measured by Transwell migration assay and Matrigel‐based Transwell invasion assay, respectively, according to previously published methods [16].

### Statistical analysis

All values were expressed as mean ± standard deviation (SD). The differences among groups were analyzed with one‐way ANOVA (for more than two groups) or Student’s *t* test (for two groups) in graphpad prism 5.0 (GraphPad Software, San Diego, CA, USA). *P* < 0.05 was considered to be statistically significant.

## Results

### The effects of TTR on blood pressure and urinary protein in l‐NAME‐induced hypertension in pregnant rats

**Fig. 1:**
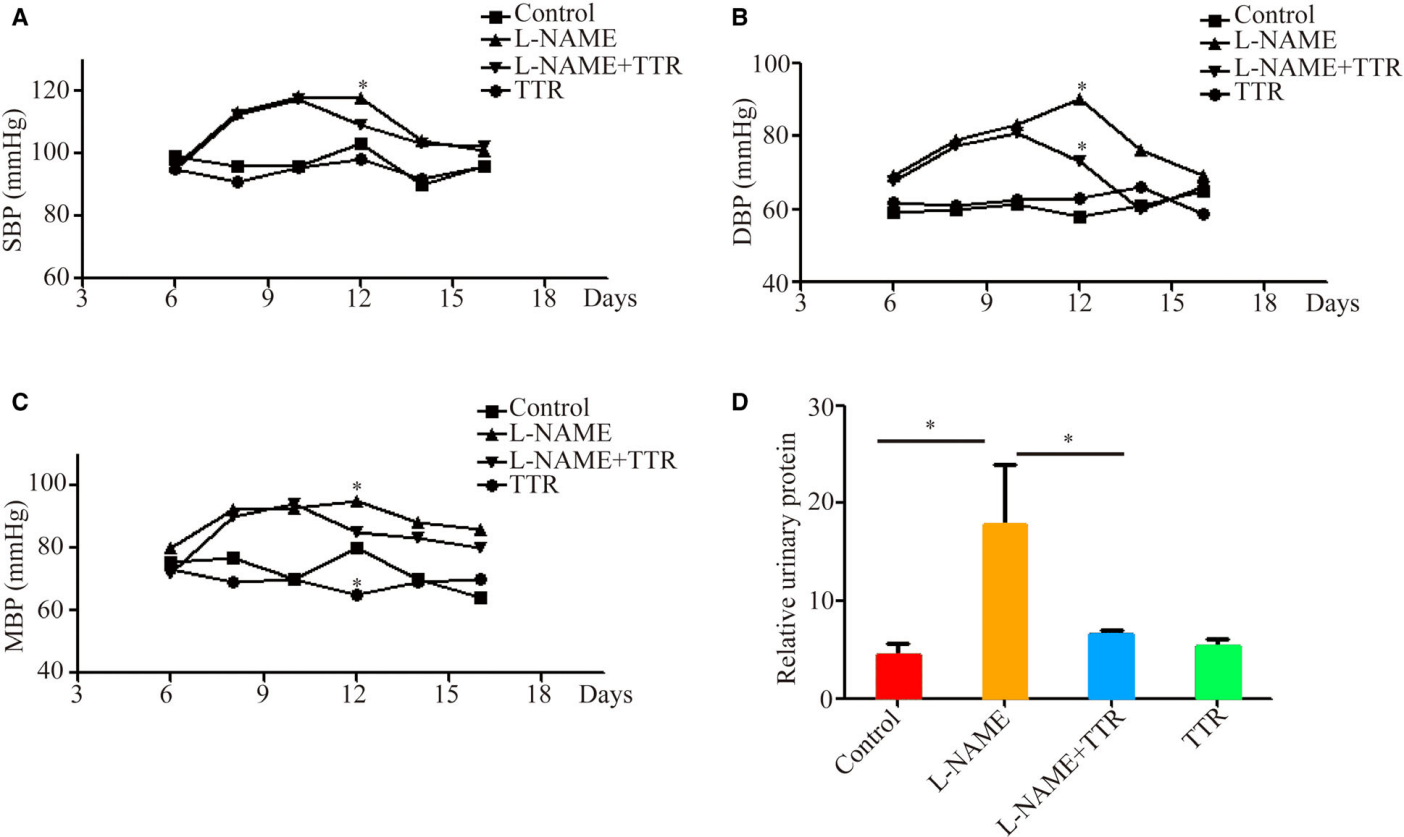
Detection of SBP (A), DBP (B), MBP (C) and urinary protein (D) in the healthy pregnancy group (control group) and pregnancy‐induced hypertension model group (l‐NAME group) treated with or without TTR. *P < 0.05, compared with control or l‐NAME. The error bars indicate SD. There were eight rats in each group.

The hypertension in pregnant rats was induced by subcutaneous injection of l‐NAME. As shown in Fig. 1A–C, the systolic blood pressure (SBP), diastolic blood pressure (DBP) and mean blood pressure (MBP) of pregnant rats in the l‐NAME group were significantly higher than those of healthy pregnancy rats (control group), suggesting the animal model was successfully established. Next, we investigated the effects of TTR on blood pressure of pregnant rats. After TTR treatment on the 11th day of pregnancy, it was found that SBP, DBP and MBP of hypertensive rats were significantly decreased compared with l‐NAME treatment alone (*P* < 0.05). In addition, there was no significant change in blood pressure between the control group and the TTR group (healthy pregnant rats with TTR treatment).

Furthermore, urine samples of each group were collected on the 17th day of gestation to analyze the urinary protein. The results showed that urinary protein in the l‐NAME group was significantly higher than that in the control group. After TTR treatment, urinary protein in the l‐NAME + TTR group was significantly reduced compared with that in the l‐NAME group, indicating that TTR might reduce the risk for albuminuria induced by pregnant hypertension (Fig. 1D).

### The pathological changes in the placenta

**Fig. 2:**
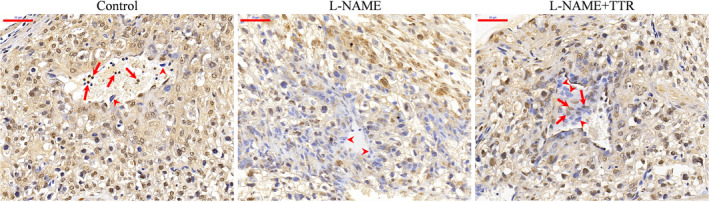
Cytokeratin 7 immunohistochemical staining to demonstrate the alterations of spiral artery remodeling in decidua of placenta from different groups. Scale bars: 50 μm.

As shown in Fig. 2, almost all spiral arteries at the superficial epithelial decidual surface showed progressive remodeling with extravillous cytotrophoblasts invasion into the lumen (arrows) in the control group; but after l‐NAME treatment, the placenta displayed some almost unremodeled arterioles without invaded extravillous cytotrophoblasts in decidua basalis. What is more, decidual veins of l‐NAME‐treated placenta possessed obviously fewer VSMCs (arrowheads) compared with that in the control group. When TTR treatment was finished, most of the arteries showed relatively advanced remodeling that has been relined with trophoblasts. Other expected pathological changes, such as invasion of trophoblasts into myometrium and endothelial cell swelling and disruption (data not shown), were too ambiguous to display.

### Therapeutic effects of TTR on pregnant rats with hypertension

**Fig. 3:**
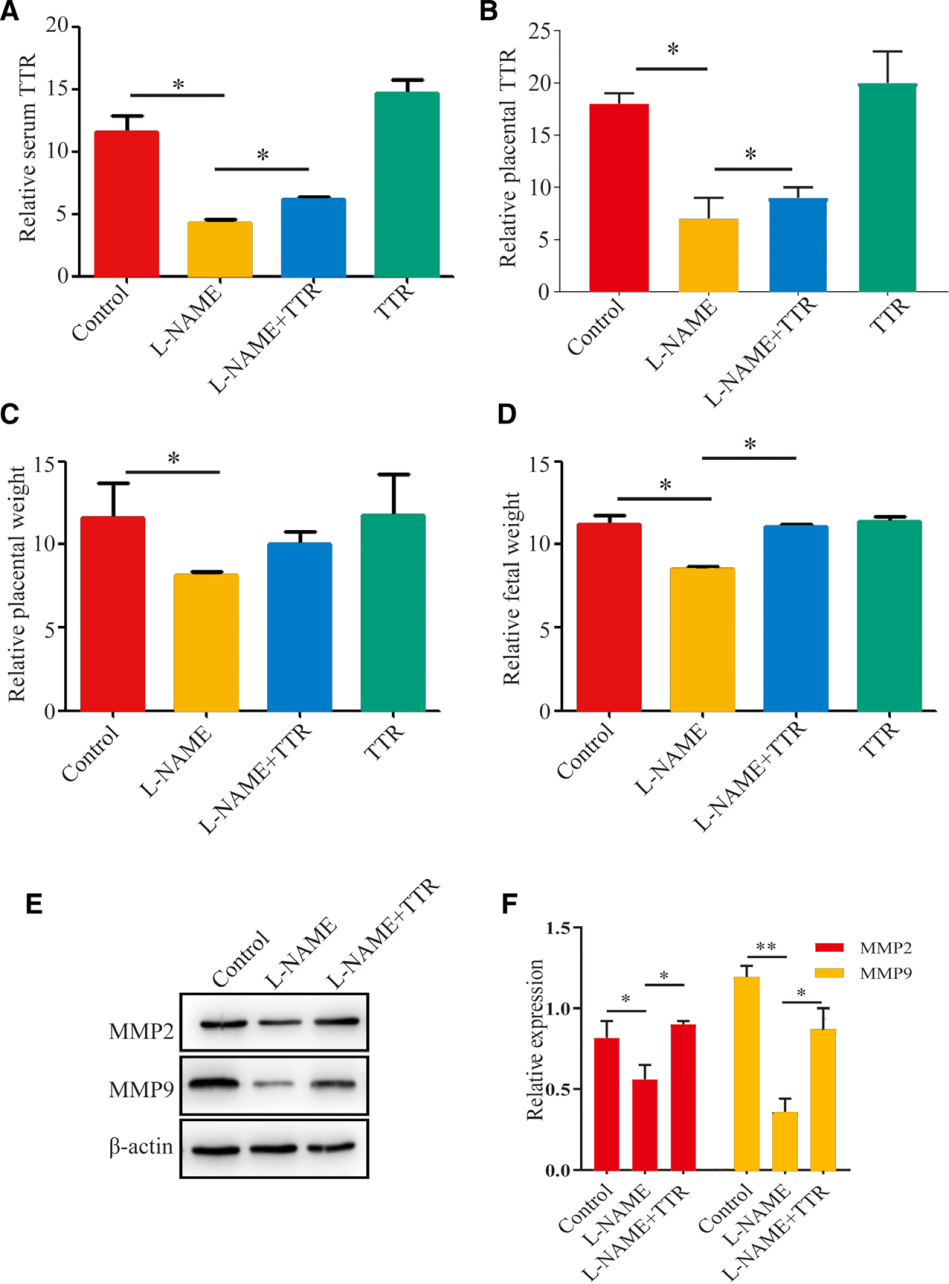
Detection of TTR protein levels in rat serum (A) and placental tissues (B) in the healthy pregnancy group (control group) and pregnancy‐induced hypertension model group (l‐NAME group) treated with or without TTR. Detection of placental (C) and fetal (D) weights in the healthy pregnancy group (control group) and pregnancy‐induced hypertension model group (l‐NAME group) treated with or without TTR. *P < 0.05. (E, F) Protein levels of MMP‐2 and MMP‐9 in placental tissues were assessed by western blotting. *P < 0.05, **P < 0.01, compared with control or l‐NAME. The error bars indicate SD. The experiments were performed independently thrice.

First, ELISA was performed to quantify the serum TTR levels (Fig. 3A) in the l‐NAME and control groups, and the results showed that TTR levels in the pregnant hypertension rats were significantly reduced compared with those in the healthy pregnant rats (Fig. 3A). The TTR levels in placenta tissues were then detected by western blot to monitor the changes of TTR during pregnant hypertension. Figure 3B shows that the expressions of TTR in the l‐NAME group (PE) were markedly decreased compared with the control group (Fig. 3B).

Furthermore, we investigated the placental and fetal weights of pregnant hypertension rats after TTR injection. Compared with the control, the placental and fetal weights in the l‐NAME group effectively were lower, but such effects could be rescued by TTR treatment in the pregnant hypertension rat model (Fig. 3C,D). To further clarify the therapeutic mechanisms of TTR on pregnant hypertension, we investigated the MMP‐2 and MMP‐9 expressions in placental tissues. The results showed that MMP‐2 and MMP‐9 levels in the pregnant hypertension rats were significantly reduced compared with those in the healthy pregnant rats, but such effects could be significantly rescued by TTR treatment (*P* < 0.05) (Fig. 3E,F).

### Rat trophoblast cells test and the expression of MMP‐2/9 in rat placental trophoblast cells

**Fig. 4:**
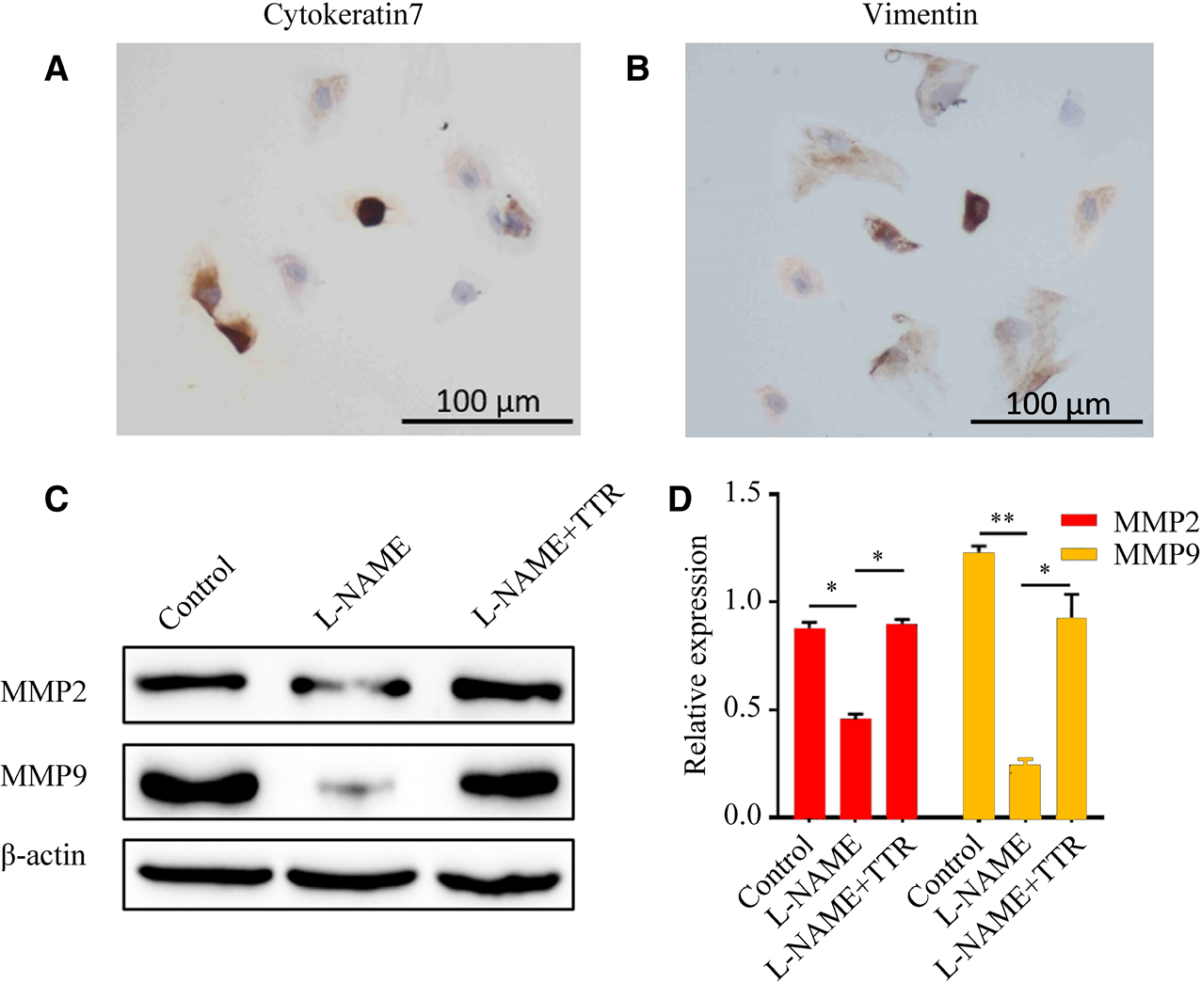
Trophoblast cell identification by Cytokeratin 7 immunohistochemistry (A) and Vimentin immunohistochemistry (B). Scale bars: 100 μm. Protein levels of MMP‐2 (C) and MMP‐9 (D) in trophoblast cells were assessed by western blotting. *P < 0.05, **P < 0.01, compared with control or l‐NAME. The error bars indicate SD. The experiments were performed independently thrice.

We used the immunohistochemical methods to observe Cytokeratin 7 and Vimentin, which were used to identify whether the isolation of rat trophoblast cells was successful. The results are shown in Fig. 4A,B. Positive Cytokeratin 7 staining reached 85%, and positive Vimentin reached 90%. To further clarify the therapeutic effects of TTR on trophoblasts, we investigated the MMP‐2 and MMP‐9 expressions in trophoblasts. The expression trend of MMP‐2 and MMP‐9 in trophoblast cells (Fig. 4C,D) was similar to that in placental tissue.

### TTR promotes the migration and invasion of rat placental trophoblast cells

**Fig. 5:**
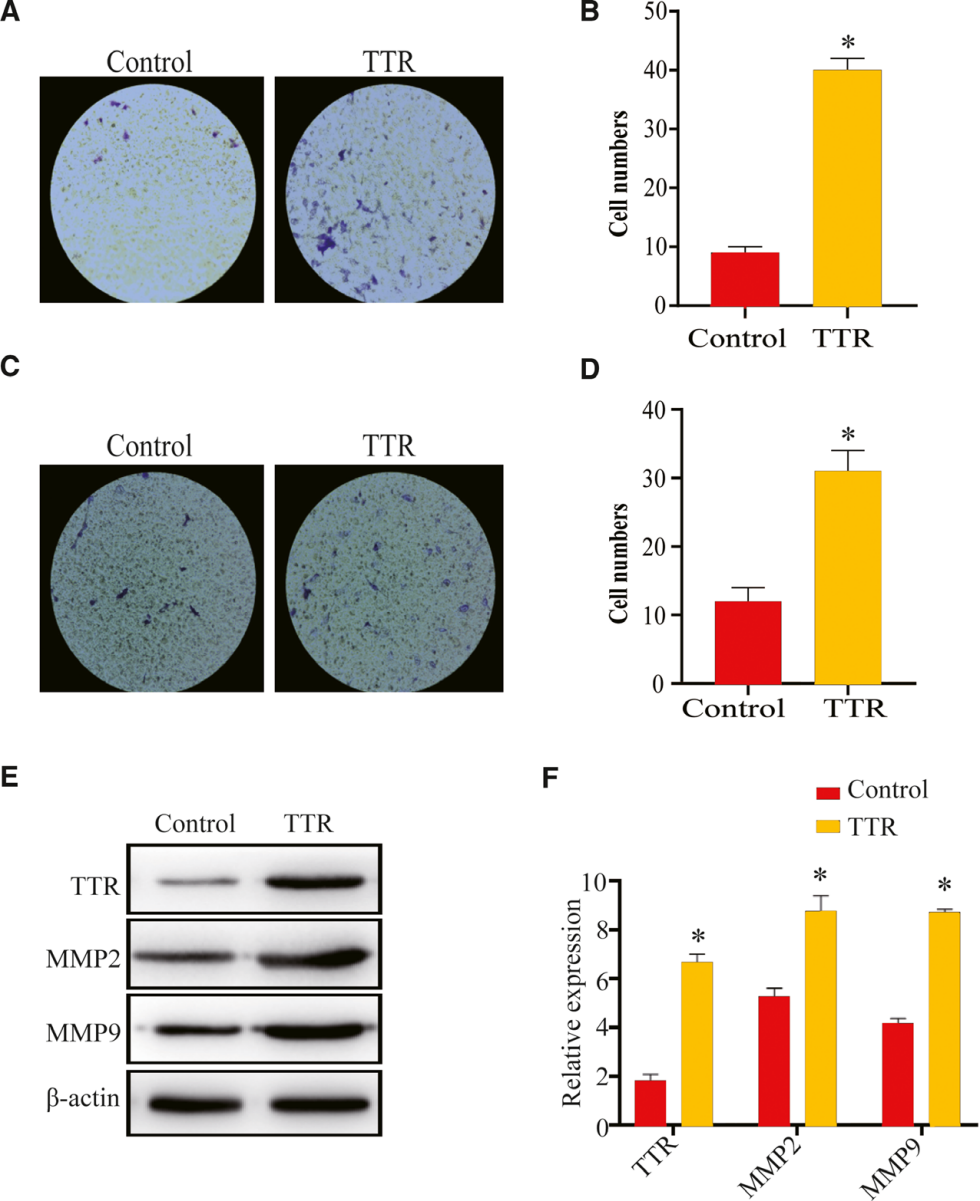
TTR promotes the migration and invasion of rat placental trophoblast cells. (A, B) Transwell migration assay was performed to analyze the migration of trophoblast cells. (C, D) Matrigel‐based Transwell invasion assay was performed to analyze the invasion of trophoblast cells. (E, F) Protein levels of MMP‐2 and MMP‐9 in trophoblast cells were assessed by western blotting. *P < 0.05, compared with control. The error bars indicate SD. The experiments were performed independently thrice.

Transwell migration assay and invasion assay were used to evaluate the effects of TTR on the migration and invasion of rat placental trophoblast cells. As shown in Fig. 5A,B, the number of invaded cells significantly increased after TTR treatment compared with the control. Similarly, the invasive potential of trophoblast cells also increased significantly after TTR treatment (Fig. 5C,D). Western blot analysis was then used to analyze the protein levels of MMP‐2/MMP‐9 after TTR treatment, and the results indicated that TTR significantly increased the MMP‐2 and MMP‐9 levels (Fig. 5E,F), suggesting that TTR is likely to regulate the migration and invasion ability of trophoblast cells via MMP‐mediated remodeling of the extracellular matrix.

## Discussion

PE is one of the common diseases that seriously threaten maternal life, with an incidence rate of about 5–12% [17]. The major reason is the decreased invasion activity of the trophoblastic cell and the dysfunction of uterine spiral artery reconstruction, which would result in the lack of oxygen and blood of placenta [18, 19]. Hypertension and proteinuria are two necessary conditions for the diagnosis of PE [20]. The pathogenesis of PE is very complicated. Here, our study focused on the thyroid hormone carrier, TTR.

TTR is a tetramer composed of four identical subunits. Each subunit comprises 127 amino acids. The main role of TTR is to participate in the transport of thyroxine and retinol [21]. Lee *et al*. [22] reported that TTR levels were increased in serum of patients with lung cancer, and TTR was able to promote tumor growth through regulating endothelial cells. Song *et al*. [23] found that the plasma TTR levels in patients with AD were significantly lower than those in healthy controls. Plasma TTR levels were negatively correlated with the pathological progression of patients with AD [23]. Other studies found that the concentration of TTR was highly increased after central nervous system injury. The plasma TTR level in the patients with poor prognosis was lower than that in the group with good prognosis, indicating that TTR also could promote nerve repair [24]. These studies demonstrated that TTR is pivotal for the diagnosis and prognosis of many diseases.

Notably, in a previous study, that TTR levels in the serum of pregnant women with PE were significantly lower than those of healthy pregnant women was confirmed [25]. An *in vitro* study showed that TTR stimulated the growth of chorionic trophoblastic cells and affected the proliferation and migration of HTR8/svneo cells [25]. In view of the particularity of ethics and pregnancy, to further study the pathogenesis of PE, we used l‐NAME to establish an animal model of pregnancy‐induced hypertension in this study. l‐NAME induced vasoconstriction by inhibiting the synthesis of nitric oxide *in vivo*, which is consistent with the pathophysiological changes during PE [26]. The results showed that the blood pressure and urine protein increased significantly after subcutaneous injection of l‐NAME in pregnant rats, which was in accordance with the clinical features of PE compared with the control group (Fig. 1). In addition, the pathological changes of placenta (Fig. 2) and the changes of placental and fetal weight further verified the reliability of the model (Fig. 3C,D). An l‐NAME‐induced pregnant hypertension rat model is effective and feasible for studying PE, and it is worth popularizing. Based on the establishment of the effective animal model of hypertensive disorders complicating pregnancy, the effects of TTR on PE were further analyzed in the next study. TTR was effective in the treatment for rat models. Our results showed that TTR could effectively reverse the increase of blood pressure and urine protein in pregnant rats with hypertension (Fig. 1). In addition, the results from ELISA (Fig. 3A) and western blot (Fig. 3B) showed that the concentrations of TTR in pregnant rats with hypertension were significantly lower than those in normal pregnant rats, suggesting that TTR might be a novel candidate biomarker for PE.

During a normal pregnancy, a series of physiological changes occur in the kidney of a pregnant woman, including slightly enlarged renal volume, increased renal blood flow, increased glomerular filtration rate, and mild hyponatremia. PE is one of the most common causes of kidney damage during pregnancy [27]. This study found that the placental and fetal weights increased after TTR treatment in pregnant hypertension rat models (Fig. 3C,D). In a word, these results indicate a good prospect of TTR for the treatment of PE.

According to a previous study [28], both total and aggregated TTR were presented in higher levels in preeclamptic placentae compared with normotensive placentae. It is interesting that no TTR aggregation was found in the placenta at our assay. We suspect that it may be the dissociation of TTR tetramer, which leads to partially unfolded monomers that aggregate into amyloid fibrils, so we could not detect it with western blotting [29].

Currently, the molecular mechanism underlying its pathophysiology remains unknown. The spiral artery begins to remodel after 9 weeks of gestation, which is followed by an increase in the oxygen supply to the placenta. The changes in the spiral artery remodel are caused by invasive trophoblast cells. If invasion is limited, vascular remodeling fails, and uterine placental circulation is reduced. According to many reports, PE is caused by limited trophoblastic invasion, failure of vascular remodeling, and decreased blood volume in the uterus placenta [30, 31]. In our study, TTR has been proved to improve the invasion of trophoblasts to some extent (Fig. 2), suggesting a potential therapeutic role of TTR to be used in PE. MMPs are a family of proteolytic enzymes that have been implicated in extracellular matrix remodeling in the process of trophocyte invasion [32]. Importantly, we also found that TTR could significantly promote the migration and invasion of rat placental trophoblast cells (Fig. 3A,B). Western blot analysis revealed that TTR increased expression levels of MMP‐2 and MMP‐9 in both placental tissues (Fig. 3E) and trophoblast cells (Fig. 4D). These findings revealed a pivotal role for TTR in regulating trophoblast invasion and migration potential, suggesting a possible molecular mechanism of PE.

## Conflict of interest

The authors declare no conflict of interest.

## Author contributions

X‐PM and C‐DL conducted experiments and were responsible for data acquisition and manuscript writing; X‐PM, G‐MC and Z‐YZ were responsible for data interpretation and data analysis; G‐MC and C‐DL helped with statistical analysis; Z‐YZ conceived and designed the study, and revised the manuscript critically for important intellectual content. Each author read and approved the final manuscript.
